# Roles and behaviours of diligent and dynamic healthcare boards

**DOI:** 10.1177/0951484819887507

**Published:** 2019-11-14

**Authors:** Naomi Chambers, Judith Smith, Nathan Proudlove, Ruth Thorlby, Hannah Kendrick, Russell Mannion

**Affiliations:** 1 Alliance Manchester Business School, University of Manchester, Manchester, UK; 2 University of Birmingham, Birmingham, UK; 3 The Health Foundation London, London, UK; 4 School of Health and Social Care Colchester, University of Essex, Essex, UK; 5 Health Services Management Centre, University of Birmingham, UK

**Keywords:** boards, governing bodies, healthcare, NHS, public sector governance, senior leadership behaviours

## Abstract

Variation persists in the quality of board-level leadership of hospitals. The consequences of poor leadership can be catastrophic for patients. The year 2019 marks 50 years of public inquiries into healthcare failures in the UK. The aim of this article is to enhance our understanding of context-specific effectiveness of healthcare board practices, drawing on an empirical study of changes in hospital board leadership in England. The study suggests leadership behaviours that lay the conditions for better organisation performance. We locate our findings within the wider theoretical debates about corporate governance, responding to calls for theoretical pluralism and insights into the effects of discretionary effort on the part of board members. We conclude by proposing a framework for the ‘restless’ board from a multi-theoretic standpoint, and suggest a repertoire specifically for healthcare boards. This comprises a suite of board roles as conscience of the organisation, sensor, shock absorber, diplomat and coach, with accompanying dyadic behaviours to match particular organisation aims and priorities. The repertoire indicates the importance of a cluster of leadership practices to fulfil the purposes of healthcare boards in differing, complex and challenging contexts.

## Introduction

The public sector board has social performance and the creation of public value as its two central purposes.^1^ Created for public boards, Carver’s policy governance model enables boards to govern by making values explicit, and by having a board member focus, in line with agency theory, entirely on ends, not on means; the latter being, in Carver’s view, the job of management. The intended effect is more authoritative boards, as well as more empowered management.^2^ The events at Stafford hospital in England are arguably an unintended effect of this position: the board there was deemed to be distant from, and largely unaware of, the daily realities in the hospital, with attention primarily on financial strategy.^3,4^

The normative position, in many jurisdictions, including in the NHS in England, since the advent of New Public Management (NPM) principles, is that public sector boards now largely follow the example set by the commercial sector.^5^ There has been some concern expressed that there can be transferability problems and issues of institutional isomorphism,^6^ in this case when governance structures and processes are copied without regard to their relevance to the public sector context.

The corporate board is used in many healthcare systems as the model of governance for public hospitals,^1^ on the basis of its combination of executive and non-executive, internal and independent, professional and lay contributions, and the requirement for diverse perspectives and skills to be brought together in order to reach collective decisions in the best interests of the organisation, its strategy, and operational accountability. In the NHS in England, particular attention has been paid to hospital boards following the report of the public inquiry into Mid-Staffordshire NHS Foundation Trust in 2013 (the Francis Report)^4^ that concluded that it was the board of the Trust that bore ultimate accountability for failings in quality and safety of care.

This paper draws on findings from empirical research undertaken in the NHS in England which sought to detect and understand changes in hospital board leadership made in the wake of the Francis Report. The motivation lies in the potential to secure generalisable insights into the ingredients of effective healthcare board roles and behaviours, drawing from existing theoretical public board governance frameworks, and empirical findings from this study. We concentrate on two research questions within that study: first, what does theory and empirical evidence from this study tell us about an appropriate range of roles for healthcare boards? Second, what are the associated behaviours connected with these roles? To address these questions, we examine the relevant literature on the role of boards within healthcare organisations, drawing on combined and complementary theoretical perspectives of board composition, roles and dynamics, with a particular focus on the interplay of these with the quality and safety of care provided by hospitals. We then go on to describe the study design and selected empirical findings related to the two questions outlined above. We structure our findings by, first, identifying variations in enactment of roles and behaviours, second, outlining the impacts and effects of board practices, and third, surfacing enablers and barriers to improving leadership. We conclude by proposing a set of theoretically informed roles for diligent, dynamic and restless boards in the healthcare sector and a repertoire of board behaviours that are connected with these roles, to inform a refreshed theoretical framework for healthcare board leadership practice. We start by outlining the healthcare board governance context, with specific reference to the NHS.

## Boards in healthcare organisations and in the NHS

In the healthcare setting, there are multiple stakeholders including patients, staff, healthcare professions, regulators, funders and government. This constrains the power of the board both in relation to setting strategy, and in monitoring and improving performance, but does not absolve it from responsibility for assuring safe, high-quality and effective patient care.

There are various structures in different countries in use for hospital boards, including non-executive trustee-style boards, unitary boards, two-tier boards and four-part governance arrangements. Under the influence of NPM (see for example, Ferlie et al.^5^), these structures have generally changed to mirror more closely the private sector style board, and with stronger devolved accountability.

In the case of England, since 1990 NHS hospital boards have been modelled on the private sector unitary board concept, with around 11 to 14 members including a non-executive chair, chief executive, executive directors and a majority of appointed (independent) non-executive directors (NEDs); all members of the board have collective responsibility for hospital performance.

Following the extensively documented failings of care at the Mid-Staffordshire Hospitals NHS Foundation Trust, Robert Francis QC identified five main areas on which all NHS organisations needed to focus, led by their boards, namely: ensuring fundamental quality standards; enabling a culture of openness, transparency and candour; having a comprehensive set of nursing standards; patient-centred leadership; and making good use of timely information to assess performance.^4^

## Theoretical framework

It has been argued that behaviours can be related to the sometimes unconscious alignment of individual members to the different theories about corporate governance.^7^ These theories have been well-rehearsed elsewhere.^8–13^ Agency theory, which is based on the belief that shareholders’ or stakeholders’ interests are likely to be at variance with managers’ interests, is associated with a challenging and defending set of behaviours in the boardroom. Stewardship theory, on the other hand, with its notion of a shared and common agenda, puts a premium on a high trust and collaborative style of working. In resource dependency theory, the main job of the board is to maximise the benefits of external dependencies which favours outward looking and ambassadorial-type behaviours. From a stakeholder theory perspective, the importance of the representation of different communities of interest is prioritised, with a resulting consensus-building behavioural orientation. Finally, in relation to the sources and use of board power, managerial hegemony posits that in practice, the managers, sometimes in combination with or responding to external agencies, make most of the decisions, with the rest of the board relegated to a rubber stamping role.

Recent thinking suggests that rather than one or other of the theories being, in general, superior or preferred, context and desired outcomes should guide which theory (or combination) and related mechanisms best fit the circumstances. As Erwin et al. suggest,^14^ depending on the hospital or health system’s mission or strategic goals, certain types of boards or board processes might be preferred and researchers should continue to study boards with this perspective in mind. For this study, we therefore draw on a realist-informed interpretation framework for healthcare boards as outlined in Table 1. This outlines the five different combinations for boards in relation to theories listed in the paragraph above, their contextual assumptions, the mechanisms used by boards and their intended outcomes. The framework posits that an underlying belief in the primary board purpose and the prevailing context is likely to drive the choice of mechanisms to achieve the board’s objectives and intended organisation outcomes. Realists suggest that exposing not only the mechanisms of change in an intervention, but more importantly their relationship to the context of implementation is key to the evaluation of complex programmes.^15^ We use this approach as the starting point for our search for a hospital board behaviour repertoire.

**Table 1.** A realist perspective for effective healthcare boards with the main board theoretical purpose driving the dynamics (from Chambers et al.^1^).

Theory about the purpose of the board	Contextual assumptions	Mechanism	Intended outcome
Agency	Low trust and high challenge and low appetite for risk	Control through intense internal and external regulatory performance monitoring	Minimisation of risk and good patient safety record
Stewardship	High trust and less challenge and greater appetite for risk	Broad support in a collective leadership endeavour	Service improvement and excellence in performance
Resource dependency	Importance of social capital of the organisation	Boundary spanning and close dialogue with healthcare partners	Improved reputation and relationships
Stakeholder	Importance of representation and collective effort; risk is shared by many	Collaboration	Sustainable organisation, high levels of staff engagement
Board power	Managerial hegemony; human desire for control	Use of power differentials	Equilibrium

In line with the theoretical pluralism advocated by Roberts et al.,^10^ this position proposes that having an orientation towards a particular board purpose brings with it the likelihood of a varying set of priorities, which then relate to different organisational performance outcomes,^1^ as indicated in Table 1. The gap in the literature, as exemplified by this framework, is that as it stands, there is no explicit consideration of board practices that have an influence, as mechanisms, on these different purposes and desired outcomes. Veronesi and Keasey, for example, found that the dominance of the healthcare ‘expert’ board (that is composed largely of healthcare professionals and insiders) led to behaviours that often precluded post-NPM behavioural norms of flexibility, responsiveness, listening and collaboration with wider stakeholders.^16^ Lee et al. confirm previous findings that many hospital boards are not fulfilling their expected roles as described in the literature.^17^ In an extensive US-focused review of hospital board governance research since 1990, Erwin et al. found that the occupational background of board members influenced choice of focus, behavioural dynamics and the need for training to fulfil quality of care monitoring.^14^

The work and functioning of boards is thus empirically variable not only as conditioned by composition and external forces but also by board processes and the deployment of the will and skill of individual board members, minimalist and maximalist board practices, and locked-in routines.^18–21^ This line of research from the authors listed above emphasises board effort as discretionary, with board members having a choice to work more or less hard in the enactment of their role.

Lynall et al. argue that board activities are subject to path dependency and a reflection of the relative power and influence of the CEO and external parties at the time of formation.^22^ Other work has focused on board behaviours^13,23^ and in particular ‘dyadic behaviours’,^23–25^ where apparently paradoxical issues need to be resolved by a board, and in so doing require multiple skills to enable the work of the board to be achieved in an appropriately sensitive and reflexive manner. Recent work has developed the concept of the ‘triadic’ board, where board leadership combines robust challenge of and strong support for the executive with significant engagement on the part of the board with internal and external stakeholders.^1^

**Figure 1.**
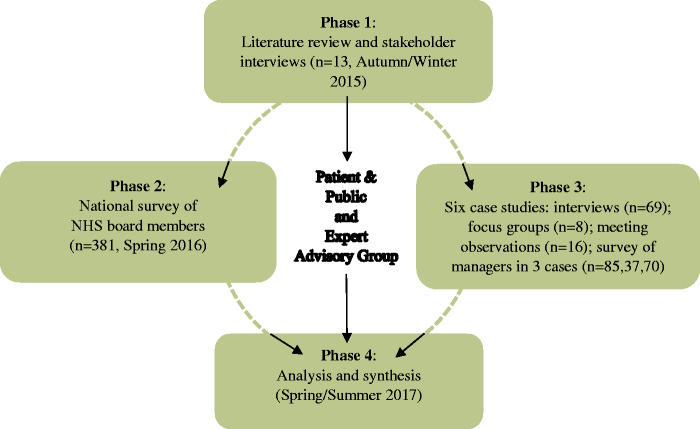
Research design.

Roberts et al. emphasised that board accountability is actualised by a whole range of behaviours that are only visible at close range.^10^ Van Puyvelde et al., for example, found that boards of nursing homes experienced a simultaneous need to both control and collaborate with their managers.^25^ They argue there is a governance tension in combining these two different roles, as it requires boards to behave in very different ways: the controlling role is more reactive and includes careful monitoring and scrutiny, and collaborating is more proactive and requires visioning and a broader understanding of the organisation and its environment. The gap in the literature is thus an understanding of the *effects* of minimalist and maximalist board practices or discretionary board effort (as described by Pettigrew and McNulty),^19^ and how dyadic cycles of supportive and controlling, trusting and challenging board behaviours can be self-correcting and lead to better organisation performance.^23^ The focus of this paper is how such greater understanding could enhance the theoretical framework for effective unitary healthcare boards (Figure 1) that is our starting point.

We now report on a mixed-methods study that sought to open up the ‘black box’ of board practices within the healthcare sector in the NHS in England.^26^ We ask, first, what does theory, and empirical evidence from this study, tell us about an appropriate range of roles for boards in the context of the NHS, and, second, what are the associated behaviours connected with these roles. Along the way, we investigated how board practices reflected the different theories (Table 1), to better understand dyadic combinations of behaviours, and possible barriers to productive combination. Our aim was to understand better the differential impact of discretionary board effort, or minimalist and maximalist board practices^19^ and how cycles of supportive and controlling, and trusting and challenging board behaviours can be self-correcting and lead to better organisation performance,^23^ and how this could enhance the proposed theoretical framework for effective unitary healthcare boards. To achieve this, the research examined the behaviours of hospital boards in relation to effective organisational leadership and shaping organisational culture, and identified ways that enable boards to prioritise patient safety (reducing the risk of harm), improving the experience of care (the collaborative effort between patient and clinician) and enhancing the clinical effectiveness of care (the capability for service improvement). All of which were key themes in the Francis Report.^4^

## Study design and methods

For the purposes of this paper, which is to advance our theoretical understanding specifically about healthcare board roles and behaviours, we report in particular on the findings in relation to these matters.

Given the intrinsic complexity of the contribution of board roles and behaviours to effective healthcare governance, the research adopted a multi-method approach, with four different phases, and integrating qualitative and quantitative elements to examine these relationships in both breadth and depth (see Figure 1). The different sources of data also enabled triangulation to take place to strengthen the development of theoretical propositions about healthcare board roles and behaviours.

To ensure that the study was grounded in the most recent theoretical and empirical governance scholarship and was cognisant of current policy developments, we carried out an initial scoping study (Phase 1) which included 13 stakeholder interviews with national-level stakeholders and opinion leaders. To capture the *breadth* of associations between recent board actions and care quality, we conducted a national survey identifying the range of measures taken by hospitals in response to recommendations in the Francis Report and subsequent national guidance (Phase 2). This survey, with 381 responses, gathered mainly quantifiable data to map connections between perceived board purposes and impacts, including barriers to action and contextual influences. To contribute *depth*, we used comparative case study methods and qualitative approaches to explore the detailed implementation and effects of recent actions of boards in six hospital trusts (Phase 3). This included a survey of ward and department managers in three of the case study trusts. The final phase was to analyse separately and then synthesise the findings from the three earlier phases to produce a set of practical, evidence-based and theoretically-informed learning points for boards.

Scoping work involved 13 interviews (four by phone and nine face to face) with key individuals from national organisations representing patients, medical and nursing professions, healthcare regulators, policy think tanks and Department of Health representatives, eliciting views on current concerns for boards, actions expected to have been taken as a result of the Francis Inquiry, the perceived and actual role of boards in overseeing and improving care quality and safety, the desirable characteristics of healthcare boards and the barriers to improving board-level leadership in the NHS. The interviews were either recorded or extensive notes taken and thematically analysed.

The purpose of the survey was to gather mainly quantifiable data about boards and how members see the board impacting on the organisation, including changes since the publication of the Francis Report in February 2013. We surveyed CEOs, chairs, chief nurses, directors of finance, medical directors, non-executive directors, and board secretaries between December 2015 and May 2016. We asked questions about:

1. Specific actions to improve board and organisational leadership (e.g. new policies, processes)
2. Perceived impacts on intermediate outcomes (e.g. organisational strategies, structures, culture) and on organisational performance
3. Perceptions of the connections between actions and impacts, including underlying mechanisms, barriers faced and contextual influences.

A mix of tick box and free text responses were sought in order to facilitate both comparative statistical analyses and an understanding of underlying issues and the influence of contextual factors. We were aware of needing to keep the questionnaire short because board members have many demands on their time and because of evidence of association between length of survey questionnaires and diminishing response rates.^27^

A total of 381 respondents completed the survey (20% response rate). At least one response was received from 139 (90%) of the 154 NHS hospital trusts and foundation trusts in England at that time.

We purposively selected six case studies using criteria for maximising the range that were agreed at the stakeholder workshop convened to refine our research approach. These included geographical variation, a mix of larger teaching hospital and smaller district hospital trusts, single and multi-site, greater or lesser stability of board membership, higher and lower performing organisations (as determined by the Care Quality Commission and Trust Development Authority assessments), foundation and non-foundation trusts and at least one specialist acute trust. We used a comparative case-study design to generalise theoretically from within and between cases.^28^ While each case has its own integrity in terms of theory building and generating policy implications, we developed common themes across sites using comparative case study methods and pattern matching.^29,30^

Data collection methods for case study work included semi-structured interviews with executive and non-executive board members of trusts, commissioners, staff representatives, patient groups and the trust board secretary. A minimum of 12 interviews took place in each case study site, supplemented by two governors, patient and staff focus group discussions or a series of individual in-depth interviews per site. We also observed one public board meeting and one meeting of governors in each site and a number of board committees, using these to inform our understanding of local board and organisational dynamics. We undertook documentary analysis of board papers, trust annual plans and reports (including about staff engagement and development, and patient and public involvement), materials related to board development activities, data on board and organisational development and quality accounts. In interviews and focus group discussions, we explored knowledge and views of board initiatives taken in response to the Francis Inquiry. These included assessments of the relevance, usefulness, and impact of such actions, costs in terms of staff and others’ time, barriers encountered and thoughts about how best to improve further the governance and leadership of the board and trust. In addition, we were able to administer an online survey questionnaire to middle managers (ward and department managers) at three out of the six case study sites. Two of the others declined to participate, and in the remaining trust, the response rate was too low for completed questionnaires to be considered for analysis. The survey was based on items in our national board-level survey. A particular aim was to elicit qualitative responses from a wider population to supplement the interviews and focus groups. Therefore, we included many free-text-response items.

All interviews and focus group discussions or notes were fully transcribed and we used qualitative coding software (*Dedoose*) to facilitate data storage and retrieval in analysis. All members of the research team were involved in generating coding frames for themes from qualitative data, and we carried out an exercise to compare independent coding of a subset of data to identify and address coding differences and ensure consistency. As part of our testing of emerging themes and for checking external validity, we offered to present our findings at a board meeting. Three case study sites responded to our invitation to feedback, thus helping us refine our final report.

## Empirical findings

We now report selected findings from the study, highlighting those results that relate to the focus of this paper. We draw on identified variations in enactment of roles and behaviours, the impacts and effects of board practices, and enablers and barriers to improving leadership, in order to propose an enhancement to current theoretical understandings of roles and behaviours for effective healthcare board governance.

### Variations in enactment of roles and behaviours

The first phase comprised interviews with key national-level stakeholders and opinion leaders. In terms of focus, there were four main areas of board working that were considered by opinion leaders to be crucial. First was a palpable concentration of effort towards ensuring patient-centred care. Second was the need to support staff, heed concerns and provide protection from negative pressures. A close alignment between what the board says and what staff say about what is going on in the organisation was a good indicator of a positive organisation culture. Third was the importance of enabling a climate for compassionate care, insisting on certain behaviours and ensuring good governance. Running through all these was the priority that should be accorded to quality, safety and learning for improvement, and, as one informant quoted from Dixon-Woods et al. ‘*more problem-sensing than comfort-seeking*’ (p.114),^31^ ensuring that the quest for assurance is balanced with a drive for improvement. Underpinning this effort, our informants told us that the board should be receiving detailed and timely data on patient and staff concerns, ensuring that quality improvement is hardwired through organisation, and using good quality data and information as the basis for improvement.

Reflecting the expectations of our opinion leader informants, the findings from the national survey (Phase 2) demonstrated the strength of ambition of NHS board members to make cultural changes in the wake of the Francis Report. This ambition was mirrored in our six case study sites (Phase 3). There was, however, variation in the extent to which these ambitions were realised. We relate the effects of this variable achievement of board aims to our conceptualisation of board leadership characteristics, practices and behaviours in the discussion section.

What contextual influences accounted for some of these differences? Variations in the perceived quality of middle management, teamworking, the embeddedness of quality improvement, the stability of board membership, and the self-perceived strategic competence of the board were connected in three of the case study organisations which were on an improving trajectory, in regulatory terms. In two of these cases, there were also reported concerns about the robustness of governance systems and processes. This indicates the sustained hard work, over an extended period of time, that is involved in the leadership effort to take organisations out of trouble. The discussion section below offers further insights into the effects of these and other, different, board leadership characteristics and behaviours.

### Impacts of board leadership practices and behaviours

In the national survey and case studies, many respondents mentioned the influence of the Francis Report in changing the focus of their board’s attention:

*It became ‘OK’ to talk about the patients and their care much more, the old adage of strategy as being the ‘in’ thing was actually eaten by the understanding that the right culture is what is really important. Looking after your patients but equally looking after your staff, communication, engagement, empowerment were all important previously, however post Francis this was ‘accepted’ as what we must do and it was not optional.* (Chief Nurse)

There were limits to board adherence to the ethos of patient-centredness of care, however. Although the average scores are high for knowledge about patients and their families, and about staff, respondents felt they had the most knowledge about what was important to regulators (Wilcoxon signed rank test p < 0.01). The proposed board role of being the conscience of the organisation which we outline in more detail below draws on this finding.

In relation to knowing what mattered to hospital staff, we found much effort had been invested in improving staff engagement. Sometimes the starting point was low, as one medical director remarked, when s/he first came into post ‘*Nobody told them they were good at anything*’. The reported impact of these efforts varied across our case study sites. One of the inhibiting factors was the empowerment of the middle-management cadre. In some of our case study sites, the onus lay too heavily on the executive tier. A further issue commented on by some staff was a lack of discipline and consistency in internal governance arrangements, accompanied by erratic internal communications. Two particular characteristics constituted excellent staff engagement, as evidenced by feedback from managers and staff: a comprehensive staff health and wellbeing strategy, and opportunities for listening and training events which successfully included the whole workforce. Evidence from the ward and department managers survey conducted at three of our case study sites suggests that a comprehensive people strategy remained somewhat of an aspiration. In particular, middle-managers’ perceptions of the opportunities for training and development, and encouragement to innovate in their trust, were very varied.

It was observed that part of the job of the board is ‘*to filter all the nonsense that comes from outside*’ (Director of Organisational Development). This interviewee, and others, felt that the board was effective in conveying to staff the importance of carrying on with caring for patients, and putting to one side some of the policy ambiguity that might be reigning in the wider NHS. This ‘shock absorber’ role of the board is developed further in our discussion section below. At the same time, there was evidence in a couple of the trusts with more stable board membership, that as well as a steady internal focus on quality, attention was paid to developing productive relationships with commissioners (local purchasers), and other local healthcare providers. For one of the trusts, which had recently come out of a regime of special measures imposed by the regulators, the board role was described as getting the basics right, a good line of sight from board to ward, and then beginning to focus on organisational strategy.

We observed in the case studies, that in order to gain assurance and promulgate core values around patient-centred care and the importance of staff engagement, the boards carried out a lot of direct communication with employees and what the Chair of the board in one hospital called ‘*dawn raids*’ to find out what has not been fixed. These efforts were generally appreciated: ‘*Some but not all of the Board are very adept at reflecting and modelling the values of the Trust in their leadership style and behaviours*’ (First-line manager); ‘*Highly committed, very supportive and focused in quality improvement, responding to risks and development of services*’ (Hospital consultant).

The longer-serving and more stable boards in our case studies exhibited greater unity and collective effort in terms of their behaviours. This was described by board members as being on the same side, and building close relationships with the senior clinical leadership of the trust, as well as being challenging, in an interrogative rather than in a confrontational way. This was the subject of probing during a regulator’s visit: ‘*Maybe I’m over sensitive: there was a slightly veiled positioning about* “*you’re daft to trust people, we shouldn't use trust as a currency*,” *whereas I always thought exactly the opposite*’ (Medical Director).

We found in the case studies that challenge by NEDs was expected, especially from the more recent appointments, and generally welcomed by executive directors. A view was expressed that they could be even more testing. Chairs were keen to coach NEDs to be appropriately challenging and in one example played devil’s advocate to provoke the expression of alternative perspectives.

One of the public board meetings observed was very stage managed, with no questions from the public and little cross-questioning, but it was directly followed by a meeting of governors at the same organisation in which executives fielded a wide range of questions. A board meeting at another trust was quite low energy and formal with little challenge from NEDs, and the meeting at a second also demonstrated fairly low challenge from NEDs. The discussions at the board meetings of the other three organisations were more spontaneous and spirited, but challenge was nearly always congenial and supportive.

We observed strong nurse leadership in four out of six cases, both internally and, to some degree, externally focused. It was suggested that the re-ordering of priorities (and board agenda items) since Francis, with a greater emphasis now on quality of care, had provided the opportunity for the chief nurse to take up a more visible and prominent role as a trust leader. As the chief nurse at one hospital put it, her role is ‘*pricking the conscience of the board continuously*’. We also observed variable contribution of executive directors beyond their functional role (for example, finance directors commenting on issues arising from the patient story). These contributions had a marked impact and other board members listened carefully. Otherwise, contribution at board meetings by executive directors was generally dependent on the board agenda item. Actively supportive relationships between medical directors and chief nurses were noted – when examples of this occurred, they enhanced messages to the board about quality and safety. The chair and CEO in all case study sites set a tone that was calm, inclusive and thoughtful. In most cases, the chair was also careful to draw in contributions from all board members and encourage executive director challenge as well as asking questions of their own. In one case, the chair tended to summarise the agenda topic rather than to invite contributions.

Using the national survey data, we conducted exploratory bivariate and multivariate regression analyses to get a sense of relationships between board practice and impact variables. We focused only on highly statistically significant relationships which are robust to the exclusion of outliers and high leverage datapoints. There were significant correlations (p < 0.01) between the amount of leadership development the board had participated in and the perceived impact of the board in relation to improving patient experience, staff engagement and patient voice.

**Figure 2.**
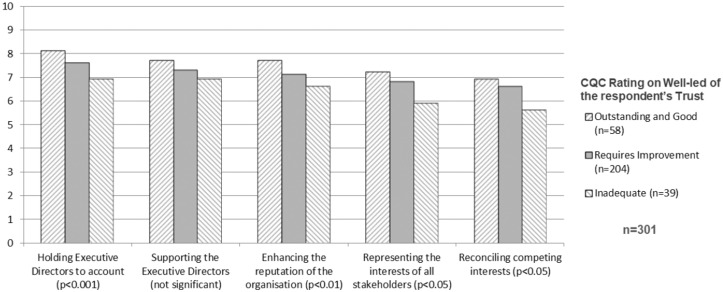
Association between averages of board members’ self-reported emphasis on different purposes and the CQC well-led rating for their Trust. (Emphasis on each purpose scored 0–10, with anchor points: 1 = Hardly at all; 3 = A little; 5 = Moderately; 7 = Quite a lot; 9 = Massively).

As described earlier, the regulator (the CQC) inspects and rates hospital trusts. The ratings are at one of four levels: Outstanding, Good, Requires Improvement, or Inadequate. By splitting our national survey responses by the rating of each respondent’s trust (combining the top two rating levels since there are very few Outstanding trusts), we see (Figure 2) that higher ratings are associated with a stronger self-reported emphasis on all the board purposes of our framework (Table 1), with the biggest difference on holding executives to account. This indicates the difference that board discretionary effort and conscientiousness may make, and the importance of a rounded repertoire of board roles, which is developed further in the discussion section below.

### Enablers and barriers

The Francis Report was found, in itself, to be in general an enabler of cultural change in hospital trusts. As one NED put it in our national survey: ‘*The Francis Report has acted as a reminder of what sort of an organisation we don't want to be like, and continues to be a reminder*’. The other main enablers were seen to be organisations which had visible senior leaders, who consistently modelled behaviours that were congruent with their values, and had good governance, communications and administrative processes. A final enabler of improved leadership was the extent to which boards themselves believed that they were able to make an impact, rather than being policy victims. Those boards which exhibited a stronger internal locus of control^32^ as measured in our national survey by self-reported scores on impacts, also maintained a focus on strategy and had a stronger quality outcomes propensity.

In the national survey, the greatest challenge the board members reported was patient safety; the most often cited barrier to improving board leadership itself was financial pressures, which presented ‘*a different kind of worry*’ (national opinion leader) for boards. As well as financial problems worsening, workforce shortages and the pressures of high patient demand also increased during the course of our study. In a tough financial environment with high levels of demand on services, the ‘iron triangle’ trade-offs^33^ of quality, cost and access dominated. As one NED put it: ‘*There are no weekends or Christmas breaks in our world and the pressure to perform miracles with less funding is unabated*’.

There were also comments about the sometimes overbearing behaviours of regulators. This was described by a stakeholder interviewee as ‘*when grip becomes throttle*’. Some of the opinion leaders, respondents to the survey of board members, and participants in our case studies expressed frustration about the inconsistency of some national guidance. They also commented that behaviours exhibited by senior leaders in national planning and regulatory bodies were often incongruent with values promulgated in their own guidance documents. The effects of these behaviours, in the views of some stakeholders, was that a focus on providing evidence of compliance consumes time and energy that should be devoted to service improvement endeavours, which, in a vicious circle, limits the possibility of gaining assurance on quality of patient care in the longer term. Developing the role of the board as a shock absorber to mitigate some of this external pressure is considered below along with other roles for hospital boards.

## Discussion

There were three types of behaviours that the opinion leader interviewees, from their national vantage point, had observed and were concerned about, which they summed up as the ‘top down’, ‘powerless’ and ‘cosy’ types of boards. Facing some common challenges (finances, meeting performance targets, patient safety) and some very different ones (legacy of failures of care, geographical isolation, longstanding strong clinical and financial performance), it was striking how the board leadership of our six case study sites also exhibited very different corporate personalities. Summing them up each individually, in one word, in alphabetical order, they were: ‘classy’, ‘courageous’, ‘defiant’, ‘ramshackle’, ‘recovering’ and ‘shiny’, with the caveat that these are to give an impression of certain characteristics of the trusts, and to illustrate diversity, rather than to pass judgement. The ‘classy’ trust has pride, self-confidence, a fantastic brand, a non-executive cadre with their own distinguished careers, and is extremely focused on clinical excellence and improving staff engagement and loyalty. The ‘courageous’ trust has had opprobrium piled upon it by the media and was seen as professionally isolated, but is now regarded as an exemplar in several areas of patient and staff engagement and has built a reputation for living by its values. The ‘defiant’ trust is a local district general hospital that used to consider itself successful, was shocked by external regulatory intervention, has a strong family feel and is somewhat defensive about external criticism. The ‘ramshackle’ trust demonstrates strong commitment to values of staff engagement and improving patient experience but consistent attention to execution and to follow-through was found lacking. The ‘recovering’ trust was picking itself up after a long period of churn on the board, poor staff morale and buffeting by regulators and the media. The ‘shiny’ trust has superb administrative systems and processes, and an excellent reputation for its staff engagement strategy and for patient-centred care. This provides a granular picture of how some hospital boards in England are wrestling, more or less successfully, with trying to deliver cultural change to provide well-organised and compassionate care for patients.

### Roles of hospital boards

Our analysis of the leadership changes made by NHS hospital trust boards since 2013 leads us to highlight five key roles for healthcare boards. These roles are based on the main board mechanisms that were identified in the context-mechanism-outcome realist framework for healthcare boards (Table 1) and elaborated by identifying the behaviours, reasoning and responses of participants in our study, as suggested by Dalkin et al.,^15^ in an extended understanding of the mechanisms in our framework. We consider these five roles in turn:

The board as conscience

The board as shock absorber

The board as diplomat

The board as sensor

The board as coach

First, in relation to the role of the board as conscience of the organisation, the findings of this research underline the need for NHS boards to own the legacy of Francis in respect of upholding fundamental standards of care, and the principles of the NHS Constitution^34^ even when the external context makes it difficult to do so. This adds up to an explicit elaboration of the social performance purpose of the public board referred to earlier. This role of being the conscience of the organisation includes leading the development of a core set of values, deliberative and inclusive approaches to making priority-setting decisions, and using listening and questioning behaviours.

Second, with regard to the role of the board as shock absorber, a theme running through this study is the burden of external regulation experienced by some board members. In an often frenetic policy environment where new initiatives can appear to shower down on hospitals, boards need to act as a shock absorber for their organisation. This means absorbing the attention and challenge of multiple external bodies, probing where necessary, distilling the feedback into messages that can be used to guide and support changes, and sheltering staff from unhelpful external ‘noise’. This can include appropriately courageous behaviours when communicating with external national bodies.

Third, we identify the role of the board as diplomat, having the curiosity to understand the full range of internal and external stakeholder interests and perspectives, and knowing how to relate to other providers and operate within the local health and care economy. As a board secretary described it in the national survey:

*Although the relationships with others in the local economy could not be said to be ‘poor’, they are not necessarily helpful. What is lacking is system leadership to try to overcome individual agendas and encourage collective thinking and action for the benefit of patients.*

This also includes promoting the reputation of the organisation using ambassadorial type behaviours.

Fourth is the role of the board as sensor, and, as Dixon-Woods et al. have observed,^31^ with more of a problem-sensing than a comfort-seeking orientation when scrutinising, with skill and wisdom, an appropriate range of performance information. One of the lines of inquiry pursued in this research was that of healthcare boards needing to assume a stronger stakeholder role, engaging with others, restlessly, to find out about problems, determine solutions and seek constantly to improve care. In working with managers and staff in the pursuit of better and safer care, this can include exhibiting both challenging and supportive behaviours.

Fifth, in the turbulent times observed during our study and with the imperative for service improvement and striving for excellence to ensure sustainable and clinically effective care, it was clear that boards also had a valuable role as coach. This involves setting ambition and direction, assessing performance, and supporting staff, in an inquiring and collaborative way. We found that this role is best likely to be fulfilled when there is visibility, stability and continuity in board membership, and board members are trained and developed to deploy a wide repertoire of behaviours.

### Repertoire of board behaviours

We further propose that the five roles for healthcare boards described above are associated with certain modes of behaviour, building on the work of Cornforth^7^ who assigned particular kinds of behaviours to different board theories. We also referred in the introduction to other work that highlighted dyadic board behaviours^23,24^ where a range of issues need to be handled or resolved by a board, and in so doing require multiple skills which are constantly held in tension in order to enable the work of the board to be achieved in an appropriately sensitive and reflexive manner.

So in the context of a low appetite for risk, and with the board in its role of sensor seeking out truths about performance, and using an agency theoretical frame, the likely dominant mode of behaviour is likely to be challenging, but also supportive (particularly in view of the unitary board model, and to ensure management is not driven to hide unpleasant facts about performance). In circumstances which particularly call for a coaching role to encourage collective innovation, improvement and striving for excellence, the likely dominant behaviours will be collaborative and inquiring, drawing from a stewardship theoretical perspective. When the external environment suggests the need for building the social capital of the organisation, which relates to a resource dependency theoretical view and the board enacting its role as diplomat, behaviours which demonstrate curiosity and ambassadorship are called for. A focus on high levels of staff engagement and long-term organisational sustainability indicates the importance of representation, collective effort and sharing of risks, the board as the conscience of the organisation, with listening and questioning behaviours coming to the fore to reflect the stakeholder perspective. Finally, the board acting as shock absorber to ensure an external equilibrium of power interests will need to be both probing and courageous. These roles for healthcare boards and associated behaviours are presented in a proposed extended theoretical framework for effective healthcare boards in Table 2.

**Table 2.** Revised framework for effective healthcare board roles.

Theory about the purpose of the board	Contextual assumptions	Roles and modes of behaviour	Mechanism	Intended outcome
Agency (holding management to account)	Low trust and high challenge and low appetite for risk	Board as sensor challenging, supportive	Holding to account and control through intense internal and external performance monitoring	Minimisation of risk and good patient safety record
Stewardship (supporting management)	High trust and less challenge and greater appetite for risk	Board as coach collaborative, inquiring	Broad support in a collective leadership endeavour	Service improvement and excellence in performance
Resource dependency (enhancing the reputation of the organisation)	Importance of social capital of the organisation	Board as diplomat ambassadorial, curious	Boundary spanning and close dialogue with healthcare partners	Improved reputation and relationships
Stakeholder (representing interests of all stakeholders)	Importance of representation and collective effort; risk is shared by many	Board as conscience listening, questioning	Collaboration	Sustainable organisation, high levels of staff engagement
Board power (reconciling competing interests)	Human desire for control	Board as shock absorber Courageous, probing	Use of power differentials	Equilibrium

**Figure 3.**
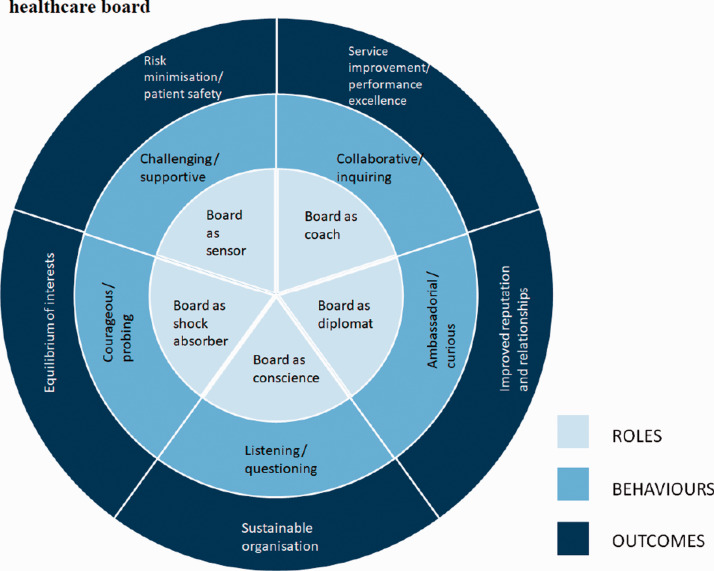
Interconnectedness of roles, behaviours and outcomes of the dynamic healthcare board.

Our revised governance framework and our proposed repertoire of board behaviours reflect the notion that the lived experiences of board members do not fit neatly into traditional theoretical governance divisions. Switching from one type of leadership behaviour to another according circumstances may be important, but so too is the ability to deploy, across time, all the roles of the diligent board, suggested by our findings about the associations between regulatory ratings and board self-reported emphasis on different purposes (Figure 3). This is akin to Roberts et al.’s creation of skilful accountability^10^ and relates also to Garratt’s natural rhythm of the board’s year and the four tasks across the 12 months (policy formulation, strategic thinking, supervising management and ensuring accountability).^35^ This study therefore suggests that the deployment of not one but a cluster of board practices, related to the full range of the main corporate governance theories, may be positively linked with organisation performance. Holding to account (agency theory) and support for managers (stewardship theory) are both important. So too is the fulfilment of other purposes of the board. The findings from the national survey suggest that the diligent board goes beyond the high-trust, high-challenge, high-engagement proposition to a fuller board repertoire including emphases on enhancing the reputation of the organisation (resource dependency theory), representing the interests of stakeholders (stakeholder theory) and reconciling competing interests (power theory). The boards of organisations with higher regulatory ratings had statistically significant higher emphasis scores on all these purposes, as reported by board members. This indicates the impacts of the minimalist and maximalist board practices alluded to by Pettigrew and McNulty^19^, Storey^21^ and others. The highest scores in this study were for holding to account, suggesting that there are dangers in taking the foot off the pedal on this purpose and the importance of the ‘restless’ board. Through depicting board activities as a wheel that turns, Figure 3 demonstrates the interconnectedness of roles, behaviours and outcomes of the restless and dynamic healthcare board.

We would argue that although this research is based on hospital trusts in England, the findings are also largely relevant for other healthcare organisations as the interpretation of empirical findings draw upon on generic corporate governance theoretical propositions. Further, although governance models and political environments in other countries may vary, the quest for high quality and safe patient care and the impact of board failings has an international resonance.

## Conclusions

This paper aims to further our understanding about an appropriate range of roles for boards in the context of healthcare, and associated behaviours connected with these roles. Our mixed-methods study examined how board leadership in NHS hospitals in England has changed in the aftermath of the Francis Inquiry into the failure of care at Stafford hospital, and has illuminated the complexity of relationships between contexts, focus, behaviours and outcomes. Our contribution is threefold. First, we offer a typology of board roles specifically for the challenging strategic and operational healthcare environment, and the need, first and foremost to focus on the provision of safe, compassionate and effective care. Second, we provide insight into the circumstances in which the enactment of roles and a repertoire of board behaviours may be conducive to certain outcomes, mindful of the call for theoretical pluralism in opening the ‘black box’ of boards. Third, we adduce further evidence for the difference that the diligent and dynamic board can make. This builds on the work of board governance scholars from Mace^18^ onwards who have exposed the gap between the myths and the realities of the work of boards, and the impact of minimalist and maximalist board practices.

There are a number of limitations in the empirical study on which this paper is based. These include a response rate from the national survey of board members of only 20%. This is mitigated by achieving 90% coverage of all acute and specialist hospitals in England, but it still means that we have to be cautious about drawing conclusions from the results. Second, there is not yet sufficient evidence to suggest that the sets of behaviours that we have described as being connected with certain board roles are complete or necessarily exclusive to those roles.

We would, nevertheless, consider that these insights constitute a work in progress towards a theoretical framework for healthcare boards. The utility of the classification of board roles of conscience, shock absorber, sensor, diplomat and coach requires further investigation. The sets of dyadic behaviours that we have proposed as being associated with these roles need further testing. Finally, an area of future research would be to investigate the impact of the composition of the board, including backgrounds, experiences, and perspectives of board members on how roles are taken up and behaviours are enacted.
